# Anti-Phytopathogenic and Cytotoxic Activities of Crude Extracts and Secondary Metabolites of Marine-Derived Fungi

**DOI:** 10.3390/md16010036

**Published:** 2018-01-18

**Authors:** Dong-Lin Zhao, Dan Wang, Xue-Ying Tian, Fei Cao, Yi-Qiang Li, Cheng-Sheng Zhang

**Affiliations:** 1 Marine Agriculture Research Center, Tobacco Research Institute of Chinese Academy of Agricultural Sciences, Qingdao 266101, China; zhaodonglin@caas.cn (D.-L.Z.); zihuafenglin@163.com (D.W.); dongqingyuxue@163.com (X.-Y.T.); 2 College of Pharmaceutical Sciences, Hebei University, Baoding 071002, China; caofei542927001@163.com

**Keywords:** marine-derived fungi, *Fusarium equiseti*, *Alternaria* sp., anthraquinones, perylenequinones, phytopathogens, antibacterial activity, antifungal activity, cytotoxicity

## Abstract

Thirty-one isolates belonging to eight genera in seven orders were identified from 141 strains that were isolated from several marine plants. *Alternaria* sp. and *Fusarium* sp. were found to be the predominant fungi. Evaluation of the anti-phytopathogenic bacterial and fungal activities, as well as the cytotoxicity of these 31 extracts, revealed that most of them displayed different levels of bioactivities. Due to their interesting bioactivities, two fungal strains—*Fusarium equiseti* (P18) and *Alternaria* sp. (P8)—were selected for chemical investigation and compounds **1**–**4** were obtained. The structure of **1** was elucidated by 1D and 2D NMR analysis, as well as high-resolution electrospray ionization mass spectroscopy (HRESIMS), and the absolute configuration of its stereogenic carbon (C-11) was established by comparison of the experimental and calculated electronic circular-dichroism (ECD) spectra. Moreover, alterperylenol (**4**) exhibited antibacterial activity against *Clavibacter michiganensis* with a minimum inhibitory concentration (MIC) of 1.95 μg/mL, which was 2-fold stronger than that of streptomycin sulfate. Additionally, an antibacterial mechanism study revealed that **4** caused membrane hyperpolarization without evidence of destruction of cell membrane integrity. Furthermore, stemphyperylenol (**3**) displayed potent antifungal activity against *Pestallozzia theae* and *Alternaria brassicicola* with MIC values equal to those of carbendazim. The cytotoxicity of **1** and **2** against human lung carcinoma (A-549), human cervical carcinoma (HeLa), and human hepatoma (HepG2) cell lines were also evaluated.

## 1. Introduction

Societal and economic development is paying more attention to health issues and food safety. Consequently, interest in the discovery of new biopesticides and drugs from natural products has also increased. Plant diseases caused by bacterial and fungal pathogens represent major constraints on crop production and cause significant losses annually. Currently, the primary control measure involves the application of conventional chemicals that are environmentally unfriendly and rapidly lose their efficacy due to the natural development of pathogen resistance [1,2]. On the other hand, there were an estimated 14 million new cancer patients worldwide in 2012, and the number is expected to rise to an annual 19.3 million cases by 2025 [3]. Furthermore, many of these cancers still lack effective agents capable of controlling them. Marine-derived fungi have gained much attention over the past two decades because of their capability to produce a large number of novel compounds (including polyketides, meroterpenoids, terpenoids, peptides, and alkaloids), many of which possess distinct bioactivities (such as antimicrobial, anticancer, antiviral, anti-inflammatory, antioxidant, and insecticidal activities) [4].

In the present study, we report the isolation and identification of marine-derived fungi, the growth inhibitory activity against plant pathogenic bacteria and fungi, and the cytotoxicity of their extracts, as well as the isolation, structure elucidation, and biological activities of the metabolites from the active extracts. Furthermore, we have also evaluated the preliminary antibacterial mechanism of alterperylenol (**4**), one of the isolated compounds.

## 2. Results and Discussion

### 2.1. Isolation, Identification, and Phylogenetic Analysis of the Marine-Derived Fungi

**Figure 1:**
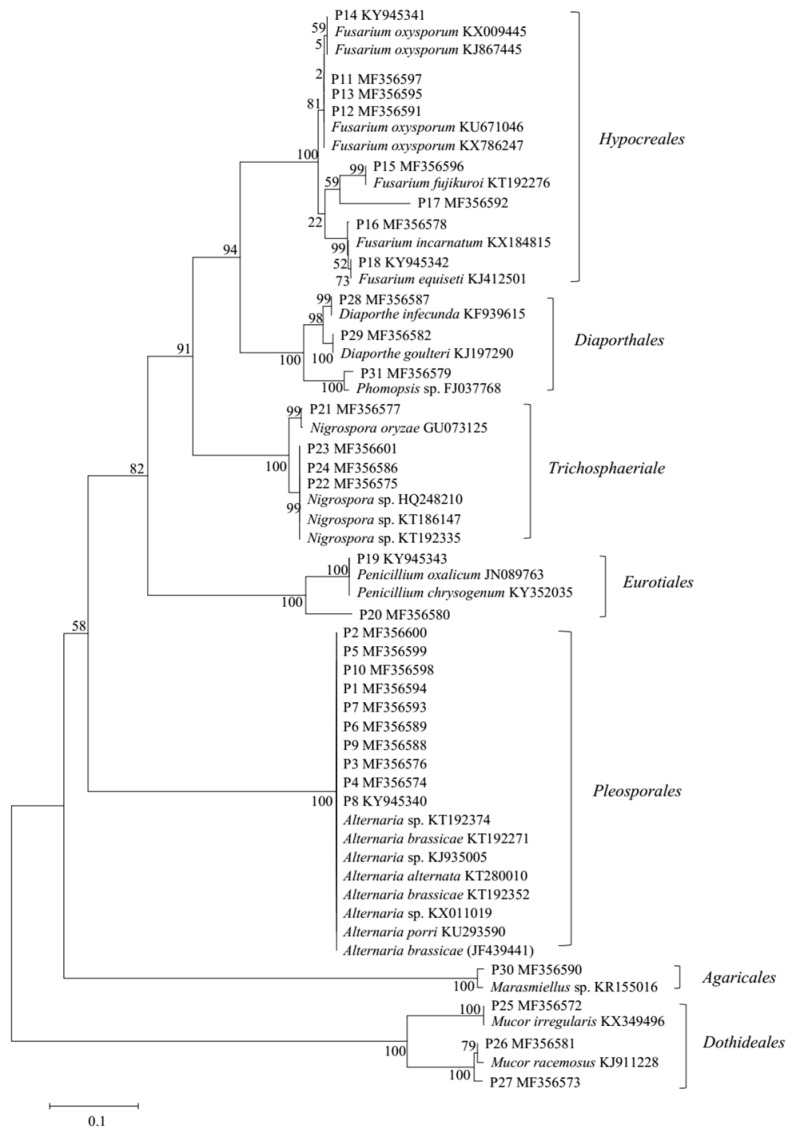
Phylogenetic tree of partial internal transcribed spacer region of the ribosomal (r)DNA gene (ITS-rDNA) sequences of marine-derived fungal strains. Reference sequences were downloaded from the National Center for Biotechnology Information (NCBI) database.

A total of 141 fungal strains were isolated from several marine plants that were collected from the intertidal zones of the Yellow Sea in Qingdao, China, and 31 strains were selected according to their morphological traits for fermentation for further chemical investigation. The 31 selected strains were preliminarily identified according to morphological characteristics and by molecular protocols that involved the amplification and sequencing of DNA from the internal transcribed spacer (ITS) region of the ribosomal (r)DNA gene (Table S1). All fungal ITS-rDNA sequences matched those of their closest relatives (99–100% similarity), as determined from the National Center for Biotechnology Information (NCBI) database. Further phylogenetic analysis was performed using MEGA 6.0 software (http://www.megasoftware.net/; Figure 1), revealing that the 31 identified fungi belonged to eight genera in seven orders, including *Fusarium* sp. (*Hypocreales*), *Diaporthe* sp. (*Diaporthales*), *Phomopsis* sp. (*Diaporthales*), *Nigrospora* sp. (*Trichosphaeriales*), *Penicillium* sp. (*Eurotiales*), *Alternaria* sp. (*Pleosporales*), *Marasmiellus* sp. (*Agaricales*), and *Mucor* sp. (*Dothideales*). Among these, *Alternaria* sp. and *Fusarium* sp. were the predominant fungi, accounting for 58.06% of those identified (10 *Alternaria* sp. strains and 8 *Fusarium* sp. strains) (Table S1).

### 2.2. Screening for Bioactive Marine-Derived Fungal Strains

#### 2.2.1. Antibacterial Activity

**Figure 2:**
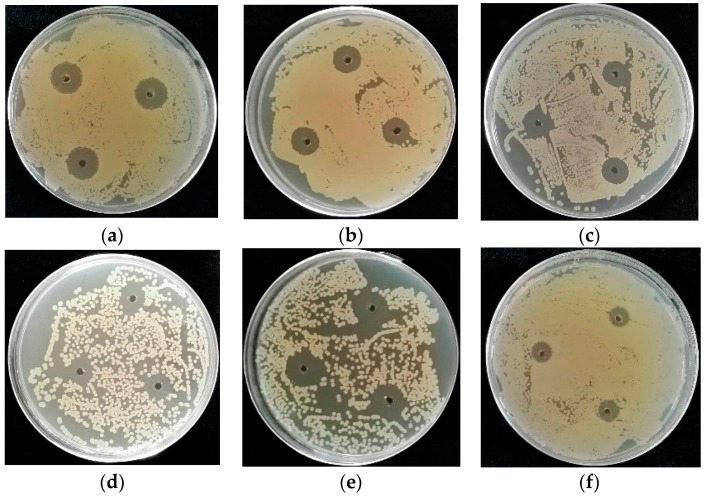
Antibacterial bioassay by the agar diffusion method. Bacterial inhibition zones of 0.1 mg/mL fungal extracts. (**a**) Extract of P18 against *P. syringae* pv. *lachrymans*; (**b**) Extract of P19 against *P. syringae* pv. *lachrymans*; (**c**) Extract of P20 against *P. syringae* pv. *lachrymans*; (**d**) Extract of P18 against *A. avenae*; (**e**) Streptomycin sulfate against *A. avenae*; (**f**) Streptomycin sulfate against *P. syringae* pv. *lachrymans*.

Most antibacterial compounds isolated from marine-derived fungi are studied for their physianthropy uses rather than agricultural applications, and few studies have focused on their anti-phytopathogenic bacterial activity. Crude extracts obtained from the fermentation broth of 31 selected marine-derived fungal isolates were evaluated for their growth inhibitory activity against plant pathogenic bacteria including *Pseudomonas syringae* pv. *lachrymans*, *Acidovorax avenae*, *Erwinia carotovora*, *Xanthomonas oryzae* pv. *oryzae*, *Ralstonia solanacearum*, and *Clavibacter michiganensis*. Most of the extracts (24; 77% of the fermented strains) showed varying degrees of antibacterial activity at 10.0 mg/mL (Table 1), whereas eight strains (26%) exhibited strong activity at 1.0 mg/mL (Table 2). The extracts of *Fusarium equiseti* (P18), *Penicillium oxalicum* (P19), and *Penicillium chrysogenum* (P20) at 0.1 mg/mL displayed stronger growth inhibitory activity against *P. syringae* pv. *lachrymans* or *A. avenae*, with P18 being the most efficient fungus (Figure 2). The marine-derived *Penicillium* sp. displayed the most potent anti-phytopathogenic bacterial activity, as both fungal strains (P19 and P20) exhibited strong antibacterial activity compared to streptomycin sulfate at concentrations of 10.0, 1.0, and 0.1 mg/mL (Figure 2). It appeared that the 31 fungal strains showed selective inhibitory activity against *P. syringae* pv. *lachrymans*, *A. avenae*, *E. Carotovora*, and *C. michiganensis* as none of the fungal isolates inhibited the growth of *X. oryzae* and *R. solanacearum* when the concentration decreased to 1.0 mg/mL. The crude extract of *Alternaria* sp. (P8) showed the broadest antibacterial spectrum since it inhibited the growth of all six tested plant pathogenic bacteria at 10.0 mg/mL.

#### 2.2.2. Antifungal Activity

**Figure 3:**
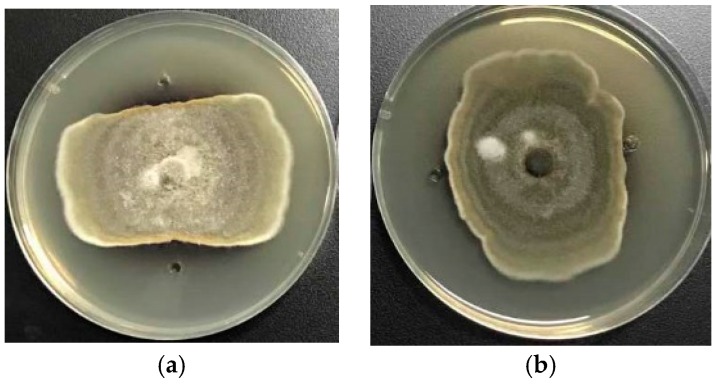

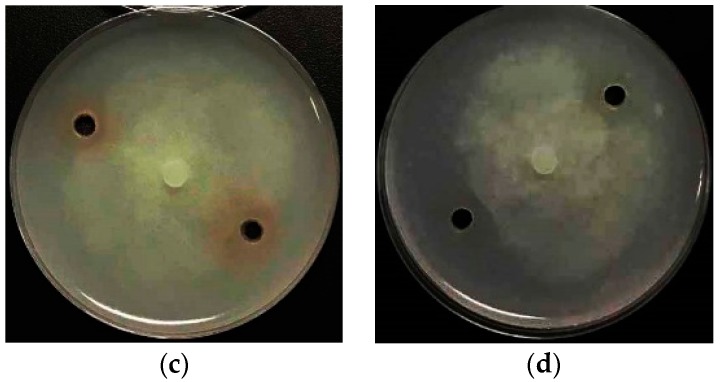
Antifungal bioassay by the Micro-Atmosphere method. Fungal inhibition zones of 1.0 mg/mL fungal extracts. (**a**) Extract of P14 against *A. alternata* (Fries) Keissler; (**b**) Extract of P29 against *A. alternata* (Fries) Keissler; (**c**) Extract of P18 against *P. parasitica* var. *nicotianae* Tucker; (**d**) Extract of P31 against *P. parasitica* var. *nicotianae*.

The 31 fungal isolates were tested for their antifungal activity against two phytopathogenic fungal strains, *Alternaria alternata* (Fries) Keissler and *Phytophthora parasitica* var. *nicotianae* Tucker. The growth of the two phytopathogenic fungi was affected by 16 marine-derived fungal extracts (Table 3). Fourteen strains were able to inhibit the mycelial growth of *P. parasitica* var. *nicotianae* Tucker, whereas only four strains restrained *A. alternata* (Fries) Keissler growth, suggesting that the fungal extracts were more effective against *P. parasitica* var. *nicotianae* Tucker relative to *A. alternata* (Fries) Keissler. This phenomenon can be explained by the fact that there are numerous *Alternaria* sp. fungi among the 31 fungal strains, and their metabolites might not inhibit fungi belonging to the same genus. P14, P18, P29, and P31 exhibited the most distinct antifungal activity as they were accompanied by clear inhibition zones (Figure 3).

#### 2.2.3. Cytotoxicity

The 31 marine fungal extracts were evaluated against a panel of tumor cell lines including human lung carcinoma (A-549), human cervical carcinoma (HeLa), and human hepatoma (HepG2). A total of 12 fungal strains (38.7%) showed cytotoxicity with inhibition rates > 50% at 50.0 μg/mL, and 10 of these strains (83.3%) exhibited low selectivity toward the three tumor cell lines, suggesting a wide anticancer spectrum (Table 4). P9, P19, P20, and P26 were the most active fungal strains with inhibition rates > 90% against all three tumor cell lines. The fungi of the genera *Penicillium* and *Mucor* displayed the most potent cytotoxicity as all five strains (P17, P19, P20, P25, and P26) showed significant inhibition rates.The genus *Penicillium*, including the marine-derived *Penicillium*, has been exploited worldwide for its biosynthetic potential for producing highly versatile cytotoxic secondary metabolites [5]. However, there are few studies focusing on the anticancer activity of metabolites from *Mucor* sp., suggesting that the three *Mucor* sp. strains described in our study might warrant further investigation. Notably, P18, which exhibited prominent antimicrobial activity, also showed distinct cytotoxicity toward all three tumor cell lines.

### 2.3. Structure Elucidation of Compounds ***1**–**4***

**Figure 4:**
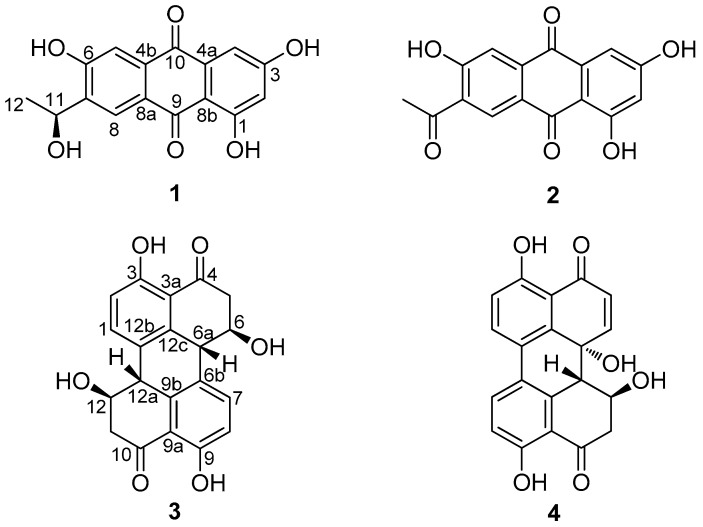
Chemical structures of compounds **1**–**4**.

Based on the results of the bioactivity screening, we performed a further chemical investigation of the P8 and P18 extracts. Two anthraquinone derivatives, (11*S*)-1,3,6-trihydroxy-7-(1-hydroxyethyl)anthracene-9,10-dione (**1**) and 7-acetyl-1,3,6-trihydroxyanthracene-9,10-dione (**2**), were isolated from the cultures of P18 and two perylenequinones, stemphyperylenol (**3**), and alterperylenol (**4**) were obtained from P8 (Figure 4).

**Figure 5:**
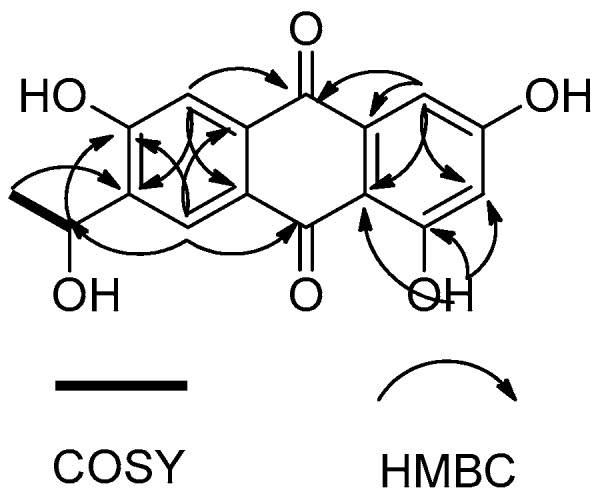
Key COSY and HMBC correlations of **1**.

Compound **1** was isolated as an orange, amorphous powder with a molecular formula of C_16_H_12_O_6_ based on the (−)-high-resolution electrospray ionization mass spectroscopy (HRESIMS) ion at *m*/*z* 299.0563 [M − H]^−^, indicating 11 degrees of unsaturation. The ^1^H NMR spectrum displayed signals for one hydrogen-bonded phenolic hydroxyl group at δ_H_ 13.04 (s), two *para*-coupled aromatic protons at δ_H_ 8.27 (s) and 7.49 (s), two *meta*-coupled aromatic protons at δ_H_ 7.09 (d, *J* = 2.0 Hz) and 6.56 (d, *J* = 2.0 Hz), one oxymethine at δ_H_ 5.01 (q, *J* = 6.5 Hz), and one methyl group at δ_H_ 1.32 (d, *J* = 6.5 Hz). The ^13^C NMR and DEPT spectra revealed the presence of two conjugated ketone carbonyls (δ_C_ 185.7 and 182.0), four methine sp^2^ (δ_C_ 125.1, 112.2, 108.1, and 107.7), one oxymethine sp^3^ (δ_C_ 62.9), eight quaternary sp^2^ carbons (δ_C_ 164.9, 164.5, 159.4, 141.0, 135.1, 133.3, 124.6, and 109.0), and one methyl group (δ_C_ 28.3). These data, in addition to correlations observed in the 2D NMR spectra (see Supplementary Materials), indicated that **1** had an anthraquinone skeleton. The HMBC correlations from OH-1 to C-1, C-2, and C-8b, from H-2 to C-4 and C-8b, from H-4 to C-2, C-8b, and C-10, from H-5 to C-7, C-8a, and C-10, and from H-8 to C-4b, C-6, and C-9 led to a confirmation of the anthraquinone nucleus (Figure 5). The COSY correlation from H-11 to H-12 and HMBC correlations from H-11 to C-6 and C-8, and from H-12 to C-7 suggested the presence of a 1-hydroxyethyl group on C-7 (Figure 5). Therefore, the planar structure of **1** was determined as 1,3,6-trihydroxy-7-(1-hydroxyethyl)anthracene-9,10-dione.

**Figure 6:**
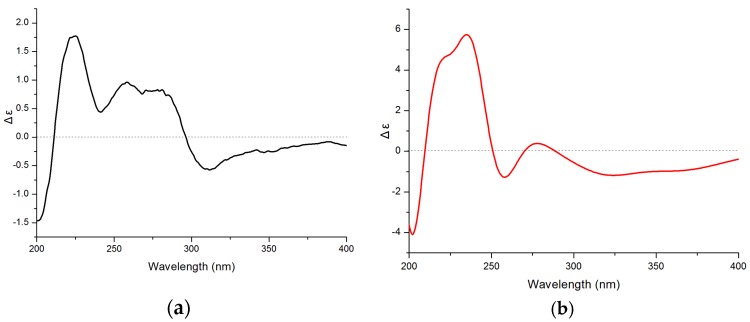
Experimental (**a**) and calculated (**b**) electronic circular-dichroism (ECD) spectra of 11*S*-**1**.

The absolute configuration of C-11 in **1** was determined by comparison of its experimental and calculated electronic circular-dichroism (ECD) spectra. First, conformational analyses were performed using the Merck Molecular Force Field 94S (MMFF94S) for 11*S*-**1**, with the results showing the 20 lowest energy conformers exhibiting relative energies between 0 and 10.0 kcal/mol. These were subsequently used in optimizations at the B3LYP/6-31G(d) level by the Gaussian09 package [6]. Subsequently, the conformers were re-optimized at the B3LYP/6-311++G(2d,p) level. In the calculated lowest-energy conformers, 10 (see Supplementary Materials) showed relative Gibbs free energies between 0 and 2.5 kcal/mol, which were used for further ECD calculations. ECD computations for all conformers were performed at the B3LYP/6-311++G(2d,p) level in vacuo, and Boltzmann statistics were performed for ECD simulations with a standard deviation of σ 0.3 eV. The experimental ECD spectrum for **1** showed first negative (312 nm), second positive (281 nm), third negative (241 nm), and fourth positive (225 nm) Cotton effects, which matched the theoretical ECD spectrum and indicated the 11*S*-configuration for **1** (Figure 6).

The stereostructure of **1** could not be found in the SciFinder database (https://scifinder.cas.org/scifinder), but the planar structure was patented without determination of the absolute configuration at C-11 or reporting accessible NMR data according to SciFinder.

(11*S*)-1,3,6-trihydroxy-7-(1-hydroxyethyl)anthracene-9,10-dione (**1**): orange powder; ${\left[\alpha \right]}_{D}^{20}$ −22.6° (*c* 0.10, MeOH); UV (MeOH) *λ*_max_ (log *ε*) 217 (4.16), 285 (4.32), 430 (3.61) nm; ECD (1.11 mM, MeOH) *λ*_max_ (Δ*ε*) 225 (Δ*ε* +1.77), 241 (Δ*ε* +0.44), 281 (Δ*ε* +0.83), 312 (Δ*ε* −0.57); ^1^H NMR (DMSO-*d*_6_, 500 MHz) δ 13.04 (1H, s, OH-1), 8.27 (1H, s, H-8), 7.49 (1H, s, H-5), 7.09 (1H, d, *J* = 2.0 Hz, H-4), 6.56 (1H, d, *J* = 2.0 Hz, H-2), 5.01 (1H, q, *J* = 6.5 Hz, H-11), 1.32 (3H, d, *J* = 6.5 Hz, H-12); ^13^C NMR (DMSO-*d*_6_, 125 MHz) δ 185.7 (C, C-9), 182.0 (C, C-10), 164.9 (C, C-3), 164.5 (C, C-1), 159.4 (C, C-6), 141.0 (C, C-7), 135.1 (C, C-4a), 133.3 (C, C-4b), 125.1 (CH, C-8), 124.6 (C, C-8a), 112.2 (CH, C-5), 109.0 (C, C-8b), 108.1 (CH, C-4), 107.7 (CH, C-2), 62.9 (CH, C-11), 28.3 (CH_3_, C-12); HRESIMS *m*/*z* 299.0563 (calcd. for C_16_H_9_O_6_, 299.0561).

The other compounds—7-acetyl-1,3,6-trihydroxyanthracene-9,10-dione (**2**) [7], stemphyperylenol (**3**) [8], and alterperylenol (**4**) [9]—were identified based on their spectroscopic data and by comparison with previously reported data.

### 2.4. Bioactivities of Compounds ***1**–**4***

We then evaluated the bioactivities of the isolated compounds (**1**–**4)** for potential anti-phytopathogenic bacterial and fungal activities toward six bacterial strains (mentioned in Section 2.2.1) and six fungal strains [*A. alternata* (Fries) Keissler, *A. brassicicola*, *P. parasitica* var. *nicotianae* Tucker, *Diaporthe medusaea* Nitschke, *Aspergillus niger* van. Tiegh, and *Pestallozzia theae*]. Additionally, the cytotoxicity of **1** and **2** toward the A-549, HeLa, and HepG2 cell lines were also tested. In the antibacterial assay, alterperylenol (**4**) exhibited prominent antibacterial activity against *C. michiganensis* with a minimum inhibitory concentration (MIC) of 1.95 μg/mL, which was 2-fold higher than that of the positive control (streptomycin; MIC = 3.90 μg/mL). Compound **2** showed potent antibacterial activity toward three strains of plant pathogenic bacteria including *P. syringae* pv. *lachrymans*, *A. avenae*, and *E. carotovora* with MIC values of 3.91, 3.91, and 7.81 μg/mL, respectively; whereas **1** exhibited weak inhibitory activity against the same bacterial strains with MIC values of 15.6, 15.6, and 7.81 μg/mL, respectively (streptomycin MIC: 0.24, 0.98, and 0.98 μg/mL, respectively). In the antifungal experiment, alterperylenol (**3**) showed inhibitory activity against *P. theae* and *A. brassicicola* with MIC values of 7.81 and 125 μg/mL, respectively, which were similar to that of the positive control (carbendazim). Compounds **1** and **2** displayed moderate antifungal activity toward *P. theae* with a MIC value of 31.3 μg/mL (carbendazim MIC: 7.81 μg/mL). In the cytotoxicity assay, **1** and **2** were inactive against A-549, HeLa, and HepG2 cell lines; however, **3** and **4** were not tested for their cytotoxic activity as they were previously shown to exhibit antitumor activity [10,11].

To further confirm the antibacterial activity of **4**, its effect on the growth curve of *C. michiganensis* was evaluated (Figure S1). In the control group, the growth curve for *C. michiganensis* revealed three clear growth phases (the lag, exponential, and stabilization phases). The bacteria began to grow rapidly during the exponential phase after 8 h (lag phase), with this lasting for 12 h until entry into the stabilization phase. However, the growth of *C. michiganensis* was completely inhibited after a 30-h incubation in the presence of **4** at a MIC concentration. These results suggest that **4** exhibited strong *in vitro* antibacterial activity against *C. michiganensis*.

### 2.5. Antibacterial Mechanism of ***4***

To investigate the mechanism(s) associated with the antibacterial activity of **4**, nucleotide leakage was evaluated and transmission electron microscopy (TEM) and membrane-potential assays were preformed to investigate the effect of **4** on the cell membrane of *C. michiganensis*. Cell-membrane destruction results in the release of intracellular components including small ions, nucleotides, and proteins, thereby making these components good indicators of membrane integrity. Free-nucleotide concentration can be estimated by assessing the optical density at 260 nm (OD_260_) and this method is widely used to determine cell membrane integrity [12]. In this study, the OD_260_ values associated with *C. michiganensis* treated with different concentrations of **4** fluctuated around 3.75 following a 30-h incubation and were similar to those observed in the control group. This indicates that no nucleotides were released in the presence of **4** and suggests that the *C. michiganensis* cell membrane was intact with treatment with **4**. To confirm this hypothesis, TEM was performed using **4** at 4× MIC. The data reveal that *C. michiganensis* cells retained normal morphology with intact cell walls and membranes similar to those in the control group (Figure S3). These findings suggest that **4** did not adversely affect *C. michiganensis* cell membrane integrity.

To further evaluate the effect of **4** on the functions associated with the bacterial cell membrane, the cell membrane potential was evaluated using the anionic lipophilic dye trimethine oxonol [DiBAC4(3)]. Upon cell membrane depolarization, the dye enters the cell and subsequently binds to intracellular hydrophobic sites, resulting in an increase in fluorescence intensity. In the absence of depolarization, DiBAC4(3) is extruded from membrane-hyperpolarized cells, leading to decreased fluorescence due to the low quantum yield of DiBAC4(3) in an aqueous environment [13]. *C. michiganensis* cells treated with **4** (MIC, 2× MIC, and 4× MIC) exhibited a clear decrease in fluorescence intensity when compared to the control group (Figure S4). At higher concentrations of **4**, lower fluorescence intensities were observed, suggesting that **4** caused significant cell membrane hyperpolarization and indicating an inhibitory effect against *C. michiganensis* proliferation.

Natural products frequently inhibit bacterial growth by affecting cell membrane integrity, causing DNA damage, and inhibiting macromolecular synthesis [14]. Due to the small amount of **4**, only three experiments evaluating the effects of this compound on the bacterial cell membrane were performed in this study. Therefore, future studies are required to investigate other factors related to the inhibitory mechanism of **4** against *C. michiganensis* proliferation.

## 3. Materials and Methods

### 3.1. Isolation of Marine-Derived Fungi

The marine-derived fungi in this study were isolated from several unidentified marine plant samples collected from the intertidal zones of the Yellow Sea in Qingdao, China (36°1′37′′ S and 120°28′11′′ E), in July 2016. The plant samples were immediately processed for fungal isolation and cultivation with the isolation of endophytic fungi performed as previously described [15]. The samples were first rinsed three times with sterile distilled water (SDW) to remove sediment and loosely attached microorganisms, followed by sterilization with 75% ethanol for 1 min and washed three times with SDW. Samples were cut into 0.5 cm^2^ pieces and transferred to potato dextrose agar (PDA) culture media with different salinity levels (0%, 0.5%, 1%, 3%, or 5%) and contained chloramphenicol (25 mg/mL) to inhibit bacterial growth. The samples were also cut into 1 cm^3^ pieces and aseptically ground with 2 mL SDW. The resulting homogenate was diluted with SDW (1:10, 1:100, and 1:1000), and 100 μL of each dilution was transferred to PDA culture media and evenly dispersed, followed by incubation at 28 °C for 1 to 3 weeks. Fungi were subsequently distinguished according to their phenotypic characteristics. Fungi exhibiting different phenotypes were replated several times for purification and transferred into cryogenic vials containing potato dextrose water (PDW) culture media and glycerol (*v*/*v* = 3:1). The strains were deposited at the Marine Agriculture Research Center, Tobacco Research Institute of Chinese Academy of Agricultural Sciences, Qingdao, China.

### 3.2. DNA Extraction, Polymerase Chain Reaction (PCR) Amplification, and Sequencing

Lysis buffer for microorganisms to direct PCR (Takara, Dalian, China) was used to release genomic DNA from the fungi. A small piece of fresh fungal mycelium and 50 μL lysis buffer were added to a sterile microtube for thermal denaturation at 80 °C for 15 min, followed by centrifugation at 5000 rpm to obtain a supernatant containing genomic DNA from each fungus. The resulting supernatant (5 μL) was used as a template to amplify the fungal ITS-ribosomal (r)DNA gene fragment, and the ITS sequence was amplified using the universal primers ITS1 (5′-TCCGTAGGTGAACCTGCGG-3′) and ITS4 (5′-TCCTCCGCTTATTGATATGC-3′) [16]. PCR was performed in a final volume of 50 μL comprising 5 μL DNA template, 1 µL each of the forward and reverse primers (10 mM), 25 µL 2 × *EasyTaq* PCR SuperMix, and 18 µL dH_2_O. After initial denaturation at 94 °C, the amplification reaction was performed over 35 cycles at 94 °C for 30 s, 55 °C for 30 s, and 72 °C for 1 min, followed by a final extension for 10 min at 72 °C. PCR products (5 μL) were detected by 0.1% agar gel electrophoresis at 120 V for 30 min and were submitted for sequencing. The sequencing results were compared with sequences from GenBank using the Basic Local Alignment Search Tool (https://blast.ncbi.nlm.nih.gov/Blast.cgi).

### 3.3. Phylogenetic Tree Construction

Sequences of the marine-derived fungal ITS-rDNA regions were compared with related sequences from the NCBI for identification. All fungal ITS sequences were aligned with the best n-BLAST hits from GenBank using Clustal X software (v1.83; http://www.clustal.org/clustal2/) using the default parameters [17]. The phylogenetic tree showing the highest homology with sequences amplified in this study was generated using the neighbor-joining method in MEGA 6.0 software (http://www.megasoftware.net/) combined with 1000 bootstrap replicates.

### 3.4. Fermentation of the Identified Marine-Derived Fungi and Bioactivity Screening

Three Erlenmeyer flasks containing each fungus were cultivated in solid medium (each containing 40 g raw rice and 30 mL H_2_O, and were sterilized) at 28 °C for 4 weeks. The fermented rice substrate was extracted repeatedly with EtOAc and CH_2_Cl_2_-MeOH (*v*/*v* = 1:1), and the solution was concentrated under reduced pressure to afford a residue that was extracted with EtOAc three times and evaporated in vacuo to yield the EtOAc extract. The EtOAc extract was subjected to silica gel column chromatography (CC) and eluted with EtOAc-petroleum ether (10%) to remove fatty acids, followed by elution with EtOAc to obtain the main components.

The antibacterial activity of the EtOAc extracts against six plant pathogenic bacteria, including *P. syringae* pv. *lachrymans*, *A. avenae*, *E. carotovora*, *X. oryzae* pv. *oryzae*, *R. solanacearum*, and *C. michiganensis*, was evaluated by agar diffusion [18]. Briefly, bacteria were cultured overnight at 37 °C in Luria Bertani (LB) broth and adjusted to a range of 2 × 10^5^ CFU/mL to 5 × 10^5^ CFU/mL. Bacterial suspensions (100 μL) were then spread on Petri dishes (diameter = 9 cm) containing 15 mL LB broth with agar. Three holes (diameter = 6 mm) were drilled into the agar plates 2 cm from the edge to form an equilateral triangle. The lyophilized EtOAc extracts were dissolved in dimethyl sulfoxide (DMSO) to final concentrations of 10.0, 1.0, and 0.1 mg/mL, followed by the addition of 10 μL of the solutions to each hole on the plates. DMSO and streptomycin sulfate were used as a negative control and a positive control, respectively. The Petri dishes were incubated at 37 °C for 24 h and the diameters of the clear inhibition zones surrounding the holes were measured.

The antifungal activity of the EtOAc extracts was tested using the micro-atmosphere method, with modifications [19]. A mycelial agar disk (diameter = 7 mm) from each plant pathogenic fungus isolated from a 7-day culture was placed at the center of a Petri dish containing 15 mL PDA medium. Two holes (diameter = 6 mm) were symmetrically drilled into the agar plate 1.5 cm from the edge of the dish. The dry EtOAc extracts were dissolved in DMSO to be used as stock solutions at a concentration of 1.0 mg/mL, and 10 μL of each extract solution was added to each hole on the dishes. Petri dishes were then incubated at 28 °C for 5 days with DMSO used as a negative control. Antifungal activity was evaluated after 5 days by observing the inhibition zones.

The cytotoxicity of the extracts against A-549, HeLa, and HepG2 cell lines was evaluated using the sulforhodamine B (SRB) method [20]. Cells were cultured in F-K12 and Dulbecco’s modified Eagle medium containing 10% heat-inactivated fetal bovine serum, 2 mM l-glutamine, 100 U/mL penicillin, and 100 μg/mL streptomycin at 37 °C in 5% CO_2_ atmosphere, with the culture medium refreshed every 2 days. Trypsinization and subculturing were performed after 80% of the cells were fused. Subsequently, 180 μL cell suspensions (in the logarithmic phase) were added to a 96-well plate (4000 cells/well) and incubated for 24 h. Fungal extracts (dissolved in DMSO) were added to the wells at a concentration of 50 μg/mL and incubated for 72 h, followed by fixation with 50% trichloroacetic acid and staining with SRB. The final concentration of DMSO in the medium was 0.1%. OD values were measured at 540 nm following the addition of 150 μL Tris. Adriamycin was used as a positive control and all experiments were performed in triplicate. The inhibition rate of cell proliferation was calculated as follows:

$$Inhibition\;rate\;\left(\%\right)\;=\;[({OD}_{540}\;(control\;group)\;-\;{OD}_{540}\;(trial\;group))/{OD}_{540}\;(control\;group)]\;\times \;100\%$$

### 3.5. Isolation and Structure Elucidation of ***1**–**4*** from F. equiseti (P18) and Alternaria sp. (P8)

Due to the small-scale fermentation, only two compounds were obtained from each extract. The pretreated crude extract of P18 (described in Section 3.4) was first subjected to octadecylsilyl silica gel (ODS) CC (RP18, 40–63 μm; Merck, Billerica, MA, USA), followed by step-gradient elution using MeOH-H_2_O (30–50%) and purification via Sephadex LH-20 CC (GE Healthcare, Pittsburgh, PA, USA) using CH_2_Cl_2_-MeOH (*v*/*v* = 1:1) to yield **1** (4.4 mg) and **2** (2.1 mg). The EtOAc extract of P8 (described in Section 3.4, 20 bottles) was first subjected to ODS CC and eluted with 30–50% MeOH-H_2_O, followed by separation by Sephadex LH-20 CC (CH_2_Cl_2_-MeOH; *v*/*v* = 1:1) and HPLC purification with 25% MeCN-H_2_O to yield **3** (4.1 mg) and **4** (3.6 mg).

Optical rotations were measured on a Jasco P-1020 digital polarimeter (Jasco, Inc., Easton, MD, USA), and UV spectra were recorded on a Techcomp UV2310ΙΙ spectrophotometer (Techcomp, Ltd., Shanghai, China). ECD spectra were obtained on a Jasco J-815-150S circular dichroism spectrometer (Jasco, Inc., Tokyo, Japan). NMR spectra were recorded on an Agilent DD2 500 MHz NMR spectrometer (500 MHz for ^1^H and 125 MHz for ^13^C; Agilent Technologies, Santa Clara, CA, USA) using tetramethylsilane as an internal standard. HRESIMS spectra were obtained from a Thermo Scientific LTQ Orbitrap XL spectrometer (Thermo Fisher Scientific, Waltham, MA, USA).

### 3.6. Biological Assay of the Isolated Compounds

#### 3.6.1. Evaluation of the MIC of the Bioactive Compounds

The antibacterial activity of **1**–**4** was evaluated against six strains of phytopathogenic bacteria, including five Gram-negative bacteria (*P. syringae* pv. *lachrymans*, *A. avenae*, *E. carotovora*, *X. oryzae* pv. *oryzae*, and *R. solanacearum*) and a Gram-positive strain (*C. michiganensis*). MICs were determined using a 2-fold serial-dilution method using LB broth, with modifications [21]. Briefly, 1.0 mg of each compound was dissolved in 50 μL DMSO and 950 μL SDW to obtain the stock solution at a concentration of 1.0 mg/mL. Stock solution (50 μL) and SDW (50 μL) were added to each medium to achieve serial 2-fold dilutions, followed by the addition of 50 μL of bacterial suspension (2–5 × 10^5^ CFU/mL) to each well, resulting in concentrations ranging from 0.98 μg/mL to 125 μg/mL. The final concentration of DMSO in the medium was 1%. The plates were incubated at 37 °C for 24 h and the MIC was determined as the lowest concentration at which no growth was observed. All assays were performed in triplicate. LB broth, DMSO (1%), and streptomycin sulfate were used as a blank control, negative control, and positive control, respectively.

The antifungal assay of **1**–**4** was performed using a conventional broth-dilution assay according to the National Center for Clinical Laboratory Standards recommendations [22]. Six plant pathogenic fungal strains (*A. alternata* (Fries) Keissler, *A. brassicicola*, *P. parasitica* var. *nicotianae* Tucker, *D. medusaea* Nitschke, *A. niger* van. Tiegh, and *P. theae*) were cultivated on PDW cultures and incubated at 28 °C for 72 h to 120 h. The final spore suspensions of fungi were adjusted to 1 × 10^5^ CFU/mL, and the seed culture was transferred to 96-well plates, followed by performance of the double-dilution method. Test samples (250 μg/mL stock solutions in DMSO and 2-fold serial dilutions) were transferred to a 96-well clear plate, and the spore suspensions were added to each well to achieve a final volume of 200 μL at concentrations ranging from 0.98 to 125 μg/mL. PDW culture, carbendazim, and DMSO (1%) were used as a blank control, positive control, and negative control, respectively. The plates were incubated for 72 h at 28 °C, and the MIC was defined as the lowest test concentration showing no visible growth after the incubation time.

#### 3.6.2. Cytotoxicity Assay for **1** and **2**

The cytotoxicity of **1** and **2** against the A-549, HeLa, and HepG2 cell lines was evaluated using the SRB method [20], with Adriamycin as a positive control.

#### 3.6.3. Growth Curves Associated with *C. michiganensis* in the Presence of **4**

The effect of **4** on *C. michiganensis* growth was evaluated according to a previously described method [23]. Briefly, logarithmic phase *C. michiganensis* was diluted to 1 × 10^6^ CFU/mL with LB broth, followed by the addition of sterile **4** to the diluted bacterial suspensions to achieve a final concentration of MIC. DMSO was added to *C. michiganensis* cultures as a control. Cultures were incubated at 37 °C with shaking at 150 rpm, and cell growth was monitored according to OD_600_ values every 2 h using a microplate reader (INFINITE 2000 PRO; Tecan, Männedorf, Switzerland).

#### 3.6.4. Nucleotide Leakage

The release of nucleotides into the supernatant was evaluated according to a previously described method, with modifications [12]. Briefly, logarithmic phase *C. michiganensis* cells were collected by centrifugation for 5 min at 5000 rpm, washed three times with 0.85% sterile saline, and adjusted to a final density of 1 × 10^6^ CFU/mL. Compound **4** was then added to the cell suspensions to final concentrations of MIC, 2× MIC, 3× MIC, and 4× MIC, followed by incubation at 37 °C. Cells treated with DMSO were tested under the same conditions. The mixture was filtered through a 0.22 μm membrane to remove bacterial cells, and cell growth was detected at OD_260_ every 2 h using a microplate reader (Tecan, Männedorf, Switzerland).

#### 3.6.5. Transmission Electron Microscopy (TEM)

TEM was performed to evaluate the effects of **4** on *C. michiganensis* according to previously described methods, with modifications [24]. Briefly, following incubation with **4** (4× MIC) for 12 h, *C. michiganensis* cells were centrifuged at 5000 rpm for 5 min and then washed with 0.85% sterile saline. The pellets were fixed with 2.5% glutaraldehyde for 4 h and 1% osmic acid for 1.5 h, followed by washing with 0.1 M phosphate buffer solution three times. The cells were gradually dehydrated using a graded series of acetone (50%, 70%, 90%, and 100%). After being embedded with epoxy and solidified at different temperatures, the sample was cut into thin sections (70 nm) using a Reichert-Jung ULTRACUT E machine (OPTISCHE WERKE AG, Munich, Austria), followed by staining with uranyl acetate-lead citrate for TEM (JEM1200; JEOL, Ltd., Tokyo, Japan).

#### 3.6.6. Measurement of Cell Membrane Potential

The membrane potential of bacterial cells was measured using the fluorescent probe DiBAC4(3) [25]. Compound **4** was added to bacterial suspensions (1 × 10^6^ CFU/mL) to concentrations of MIC, 2× MIC, and 4× MIC, followed by incubation at room temperature for 30 min. DiBAC4(3) dissolved in DMSO was added to the bacterial suspensions at a concentration of 5 μM, and after 5 min, fluorescence was measured at excitation and emission wavelengths of 492 nm and 515 nm, respectively, using a fluorospectrophotometer (F-4600; Hitachi, Tokyo, Japan).

## 4. Conclusions

In summary, 141 fungal strains were isolated from marine plants that were collected from the intertidal zones outside Qingdao, China. Two fungal strains were selected for chemical investigation according to the bioactivity screening of 31 identified strains, which subsequently resulted in the isolation of compounds **1**–**4**. These compounds exhibited potent antibacterial, antifungal, and/or cytotoxic activities, with **4** specifically being capable of causing membrane hyperpolarization in *C. michiganensis* without altering cell membrane integrity. The antibacterial activity of marine-derived fungi in this study was very interesting as there are few studies reporting the discovery and evaluation of the antibacterial efficacy of compounds from marine-derived fungi against plant pathogenic bacteria. Our results suggest that searching for compounds from marine-derived fungi that exhibit pharmacological and biological activities represent a promising strategy for identifying significant sources of potential biopesticides and drugs with novel structures and notable bioactivities.

## Figures and Tables

**Table 1:** Antibacterial activity of the extracts (10.0 mg/mL) from the culture filtrate of the marine-derived fungi.

Strain	*P. syringae*	*A. avenae*	*E. carotovora*	*X. oryzae*	*R. solanacearum*	*C. michiganensis*
P1	+	−	+	−	−	−
P2	+	+	+	−	−	−
P3	+	+	++	−	−	+
P4	++	++	++	−	−	+
P7	−	−	+	−	−	−
P8	+	+	++	+	+	+
P9	++	++	+	−	−	++
P10	−	+	++	−	−	+
P12	−	−	−	−	+	−
P13	+	+	++	−	−	+
P14	++	+++	++	−	−	++
P15	−	+	−	+	−	−
P16	+	+	+	−	−	−
P17	−	−	−	−	−	+
P18	+++	+++	+++	−	−	+++
P19	+++	+++	+++	−	−	++
P20	+++	+++	+++	−	−	++
P23	−	++	−	−	−	−
P24	−	+	+	−	−	+
P25	++	+++	++	−	+	++
P27	++	+++	++	−	−	++
P28	+	+	+	−	+	−
P29	+++	+++	+++	−	−	++
P31	+	−	+	−	−	+

Positive control: streptomycin sulfate (1.0 mg/mL); negative control: dimethyl sulfoxide (DMSO); – no antibacterial activity; + weak inhibitory activity (inhibition zone between 5 and 10 mm); ++ moderate inhibitory activity (inhibition zone between 10 and 15 mm); +++ strong inhibitory activity (inhibition zone > 15 mm).

**Table 2:** Antibacterial activity of the extracts (1.0 mg/mL) from the culture filtrate of the marine-derived fungi.

Fungal Strain	*P. syringae*	*A. avenae*	*E. carotovora*	*C. michiganensis*
P8	+	−	−	−
P14	++	−	++	+
P18	+++	+++	+++	+++
P19	+++	−	++	+
P20	+++	−	−	−
P25	+	−	−	−
P27	+	−	−	−
P29	+	−	−	−

**–** No antibacterial activity; + weak inhibitory activity (inhibition zone between 5.0 and 10.0 mm); ++ moderate inhibitory activity (inhibition zone between 10.0 and 15.0 mm); +++ strong inhibitory activity (inhibition zone > 15.0 mm).

**Table 3:** Antifungal activity of the extracts (1.0 mg/mL) from the culture filtrate of the marine-derived fungi.

Fungal Strain	*A. alternata* (Fries) Keissler	*P. parasitica* var. *nicotianae* Tucker
P3	−	+
P7	−	+
P8	−	+
P11	−	+
P12	+	−
P14	−	+
P17	+	−
P18	+	+
P19	−	+
P20	−	+
P22	−	+
P24	−	+
P26	−	+
P27	−	+
P29	+	+
P31	−	+

**–** No antifungal activity (no inhibition zones); + antifungal activity (inhibition zones).

**Table 4:** Cytotoxicity (inhibition rate) towards different tumor cell lines of the extracts (50.0 μg/mL) from the culture filtrate of the marine-derived fungi.

Fungal Strain	Inhibition Rate (%)		
	A549	HeLa	HepG2
P3	57.25	92.29	76.60
P9	91.87	96.84	93.11
P10	69.19	89.70	83.19
P12	92.70	88.88	87.29
P15	87.49	89.77	88.62
P19	96.06	97.43	97.77
P20	95.94	97.60	97.82
P21	87.02	97.08	94.53
P23	90.23	84.12	80.28
P24	96.07	97.56	97.86
P25	88.98	−	−
P30	−	65.29	−

**–** Inhibition rate < 50%.

## Supplementary Materials

The following are available online at www.mdpi.com/1660-3397/16/1/36/s1, Table S1: Identification and phylogenetic affiliations of the isolated marine-derived fungal strains, Figure S1: Effect of alterperylenol (**4**) on *C. michiganensis* cell growth, Figure S2: TEM images of *C. michiganensis*, Figure S3: Effect of alterperylenol (**4**) on the membrane potential of *C. michiganensis*, Figures S4 to S12: The ^1^H and ^13^C NMR spectra of compounds **1**–**4**, and the HRESIMS spectrum of compound **1**, Figure S13: Lowest energy conformers.

## References

[1] Sundin G.W., Castiblanco L.F., Yuan X., Zeng Q., Yang C.-H. (2016). Bacterial disease management: Challenges, experience, innovation and future prospects. Mol. Plant Pathol..

[2] Ray M., Ray A., Dash S., Mishra A., Achary K.G., Nayak S., Singh S. (2017). Fungal disease detection in plants: Traditional assays, novel diagnostic techniques and biosensors. Biosens. Bioelectron..

[3] Gomes N.G.M., Lefranc F., Kijjoa A., Kiss R. (2015). Can some marine-derived fungal metabolites become actual anticancer agents?. Mar. Drugs.

[4] Imhoff J.F. (2016). Natural products from marine fungi-still an underrepresented resource. Mar. Drugs.

[5] Koul M., Singh S. (2017). *Penicillium* spp.: Prolific producer for harnessing cytotoxic secondary metabolites. Anti-Cancer Drugs.

[6] Frisch M.J., Trucks G.W., Schlegel H.B., Scuseria G.E., Robb M.A., Cheeseman J.R., Scalmani G., Barone V., Mennucci B., Petersson G.A. (2009). Gaussian 09.

[7] Pang X., Lin X., Tian Y., Liang R., Wang J., Yang B., Zhou X., Kaliyaperumal K., Luo X., Tu Z. (2017). Three new polyketides from the marine sponge-derived fungus *Trichoderma* sp. SCSIO41004. Nat. Prod. Res..

[8] Arnone A., Nasini G., Merlini L., Assante G. (1986). Secondary mold metabolites. Part 16. Stemphyltoxins, new reduced perylenequinone metabolites from *Stemphylium botryosum* var. Lactucum. J. Chem. Soc. Perkin Trans..

[9] Okuno T., Natsume I., Sawai K., Sawamura K., Furusaki A., Matsumoto T. (1983). Structure of antifungal and phytotoxic pigments produced by *Alternaria* species. Tetrahedron Lett..

[10] Xiao J., Zhang Q., Gao Y.-Q., Tang J.-J., Zhang A.-L., Gao J.-M. (2014). Secondary metabolites from the endophytic *Botryosphaeria dothidea* of *Melia azedarach* and their antifungal, antibacterial, antioxidant, and cytotoxic activities. J. Agric. Food Chem..

[11] Wang Y., Liu H.-X., Chen Y.-C., Sun Z.-H., Li H.-H., Li S.-N., Yan M.-L., Zhang W.-M. (2017). Two new metabolites from the endophytic fungus *Alternaria* sp. A744 derived from *Morinda officinalis*. Molecules.

[12] Wu Y., Bai J., Zhong K., Huang Y., Gao H. (2017). A dual antibacterial mechanism involved in membrane disruption and DNA binding of 2*R*,3*R*-dihydromyricetin from pine needles of *Cedrus deodara* against *Staphylococcus aureus*. Food Chem..

[13] Whiteaker K.L., Gopalakrishnan S.M., Groebe D., Shieh C.-C., Warrior U., Burns D.J., Coghlan M.J., Scott V.E., Gopalakrishnan M. (2001). Validation of FLIPR membrane potential dye for high throughput screening of potassium channel modulators. J. Biomol. Screen..

[14] Santos B.S., Silva L.C.N., Silva T.D., Rodrigues J.F.S., Grisotto M.A.G., Santos C.M.T., Napoleao T.H., Silv M.V., Paiva P.M.G. (2016). Application of omics technologies for evaluation of antibacterial mechanisms of action of plant-derived products. Front. Microbiol..

[15] Qin X.-Y., Yang K.-L., Li J., Wang C.-Y., Shao C.-L. (2015). Phylogenetic diversity and antibacterial activity of culturable fungi derived from the zoanthid *Palythoa haddoni* in the South China Sea. Mar. Biotechnol..

[16] Pitzschke A. (2016). Developmental peculiarities and seed-borne endophytes in quinoa: Omnipresent, robust bacilli contribute to plant fitness. Front. Microbiol..

[17] Thompson J.D., Gibson T.J., Plewniak F., Jeanmougin F., Higgins D.G. (1997). The CLUSTAL X windows interface: Flexible strategies for multiple sequence alignment aided by quality analysis tools. Nucleic Acids Res..

[18] Wang Y.-N., Shao C.-L., Zheng C.-J., Chen Y.-Y., Wang C.-Y. (2011). Diversity and antibacterial activities of fungi derived from the gorgonian *Echinogorgia rebekka* from the South China Sea. Mar. Drugs.

[19] Yangui I., Boutiti M.Z., Boussaid M., Messaoud C. (2017). Essential oils of *Myrtaceae* Species growing wild in Tunisia: Chemical variability and antifungal activity against *Biscogniauxia mediterranea*, the causative agent of charcoal canker. Chem. Biodivers..

[20] Di T.-M., Yang S.-L., Du F.-Y., Zhao L., Xia T., Zhang X.-F. (2017). Cytotoxic and hypoglycemic activity of triterpenoid saponins from *Camellia oleifera* Abel. seed pomac. Molecules.

[21] Wang W., Liao Y., Tang C., Huang X., Luo Z., Chen J., Cai P. (2017). Cytotoxic and antibacterial compounds from the coral-derived fungus *Aspergillus tritici* SP2-8-1. Mar. Drugs.

[22] Li W., Xiong P., Zheng W., Zhu X., She Z., Ding W., Li C. (2017). Identification and antifungal activity of compounds from the mangrove endophytic fungus *Aspergillus clavatus* R7. Mar. Drugs.

[23] Zhao L., Zhang H., Hao T., Li S. (2015). In vitro antibacterial activities and mechanism of sugar fatty acid esters against five food-related bacteria. Food Chem..

[24] Liu F., Wang F., Du L., Zhao T., Doyle M.P., Wang D., Zhang X., Sun Z., Xu W. (2018). Antibacterial and antibiofilm activity of phenyllactic acid against *Enterobacter cloacae*. Food Control.

[25] Sanchez E., Garcia S., Heredia N. (2010). Extracts of edible and medicinal plants damage membranes of *Vibrio cholerae*. Appl. Environ. Microbiol..

