# Supplementary material for: Anti-Phytopathogenic and Cytotoxic Activities of Crude Extracts and Secondary Metabolites of Marine-Derived Fungi
Source: Mar Drugs. 2018 Jan 18;16(1):36. doi: 10.3390/md16010036 (PMC5793084; doi:10.3390/md16010036)
Supplement: Supplementary file 1 [file marinedrugs-16-00036-s001.pdf]

marine drugs

Supplementary Materials

Anti-Phytopathogenic and Cytotoxic Activities of Crude Extracts and Secondary Metabolites of Marine-Derived Fungi

Dong-Lin Zhao [1,†], Dan Wang [1,†], Xue-Ying Tian [1], Fei Cao [2], Yi-Qiang Li [1,*] and Cheng-Sheng Zhang [1,*]

[1] Marine Agriculture Research Center, Tobacco Research Institute of Chinese Academy of Agricultural Sciences, Qingdao 266101, China; zhaodonglin@caas.cn (D.-L.Z.); zihuafenglin@163.com (D.W.); dongqingyuxue@163.com (X.-Y.T.)

[2] College of Pharmaceutical Sciences, Hebei University, Baoding 071002, China; caofei542927001@163.com

* Correspondence: liyiqiang@caas.cn (Y.-Q.L.); zhchengsheng@126.com (C.-S.Z.); Tel.: +86-0532-6671-5517 (Y.-Q.L.); +86-0532-8870-2115 (C.-S.Z.)

† These authors contributed equally to this work.

Received: 30 November 2017; Accepted: 15 January 2018; Published: date

List of Supplementary Materials

Table S1. Identification and phylogenetic affiliations of the isolated marine-derived fungal strains

Figure S1. Effect of alterperylenol (**4**) on *C. michiganensis* cell growth

Figure S2. TEM images of *C. michiganensis*

Figure S3. Effect of alterperylenol (**4**) on the membrane potential of *C. michiganensis*

Figure S4. ^{1}H NMR (500 MHz, DMSO-d_6) spectrum of compound **1**

Figure S5. ^{13}C NMR (125 MHz, DMSO-d_6) spectrum of compound **1**

Figure S6. HRESIMS spectrum of compound **1**

Figure S7. ^{1}H NMR (500 MHz, DMSO-d_6) spectrum of compound **2**

Figure S8. ^{13}C NMR (125 MHz, DMSO-d_6) spectrum of compound **2**

Figure S9. ^{1}H NMR (500 MHz, acetone-d_6) spectrum of compound **3**

Figure S10. ^{13}C NMR (125 MHz, acetone-d_6) spectrum of compound **3**

Figure S11. ^{1}H NMR (500 MHz, acetone-d_6) spectrum of compound **4**

Figure S12. ^{13}C NMR (125 MHz, acetone-d_6) spectrum of compound **4**

Figure S13. Lowest energy conformers

Table S1. Identification and phylogenetic affiliations of the isolated marine-derived fungal strains.

Strain	Closest identified relative	Accession number	Similarity (%)	Seq. Length (bp)	Overlap (bp)
P1	*Alternaria tenuissima*	MF356594	99	568	563
P2	*Alternaria porri*	MF356600	99	568	564
P3	*Alternaria mali*	MF356576	99	565	561
P4	*Alternaria brassicae*	MF356574	99	568	566
P5	*Alternaria brassicae*	MF356599	100	566	563
P6	*Alternaria brassicae*	MF356589	99	563	559
P7	*Alternaria alternata*	MF356593	99	555	555
P8	*Alternaria* sp.	KY945340	99	574	570
P9	*Alternaria* sp.	MF356588	99	557	553
P10	*Alternaria* sp.	MF356598	99	576	573
P11	*Fusarium oxysporum*	MF356597	100	535	534
P12	*Fusarium oxysporum*	MF356591	100	523	523
P13	*Fusarium oxysporum*	MF356595	100	515	515
P14	*Fusarium oxysporum*	KY945341	99	539	534
P15	*Fusarium fujikuroi*	MF356596	100	544	544
P16	*Fusarium incarnatum*	MF356578	100	549	545
P17	*Fusarium solani*	MF356592	100	570	551
P18	*Fusarium equiseti*	KY945342	100	529	529
P19	*Penicillium oxalicum*	KY945343	100	521	521
P20	*Penicillium chrysogenum*	MF356580	99	699	695
P21	*Nigrospora oryzae*	MF356577	99	532	528
P22	*Nigrospora* sp.	MF356575	99	530	528
P23	*Nigrospora* sp.	MF356601	99	554	545
P24	*Nigrospora* sp.	MF356586	100	544	540
P25	*Mucor irregularis*	MF356572	99	626	623
P26	*Mucor racemosus*	MF356581	99	601	600
P27	*Mucor circinelloides*	MF356573	100	617	617
P28	*Diaporthe infecunda*	MF356587	99	568	545
P29	*Diaporthe goulteri*	MF356582	100	536	536
P30	*Marasmiellus* sp.	MF356590	98	668	656
P31	*Phomopsis* sp.	MF356579	98	572	551

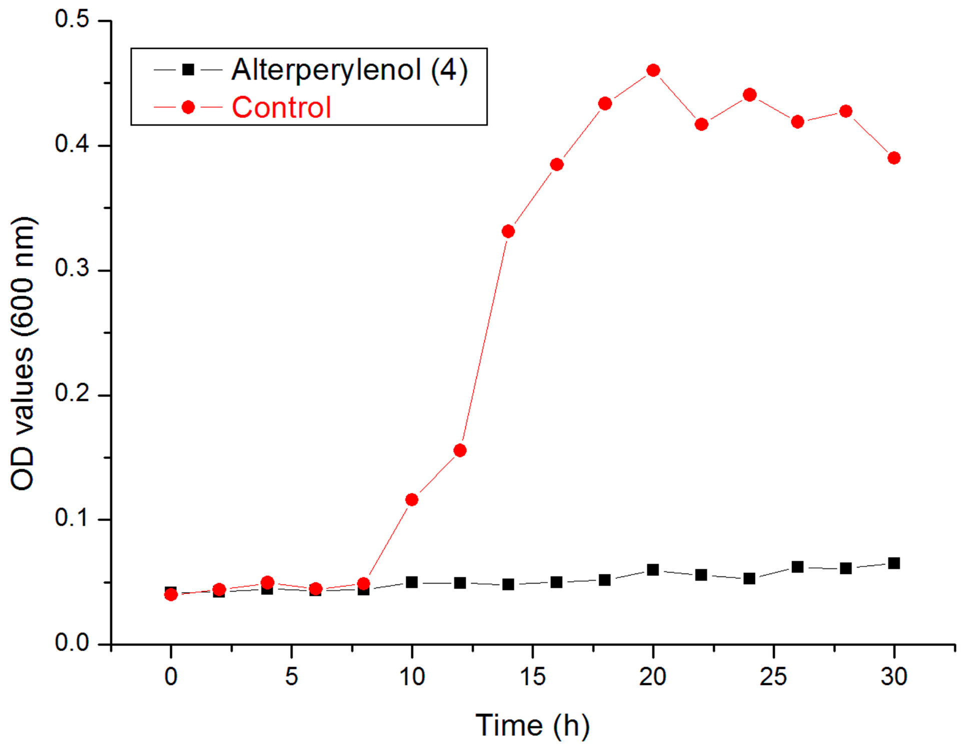

Figure S1. Effect of alterperylenol (**4**) on *C. michiganensis* cell growth

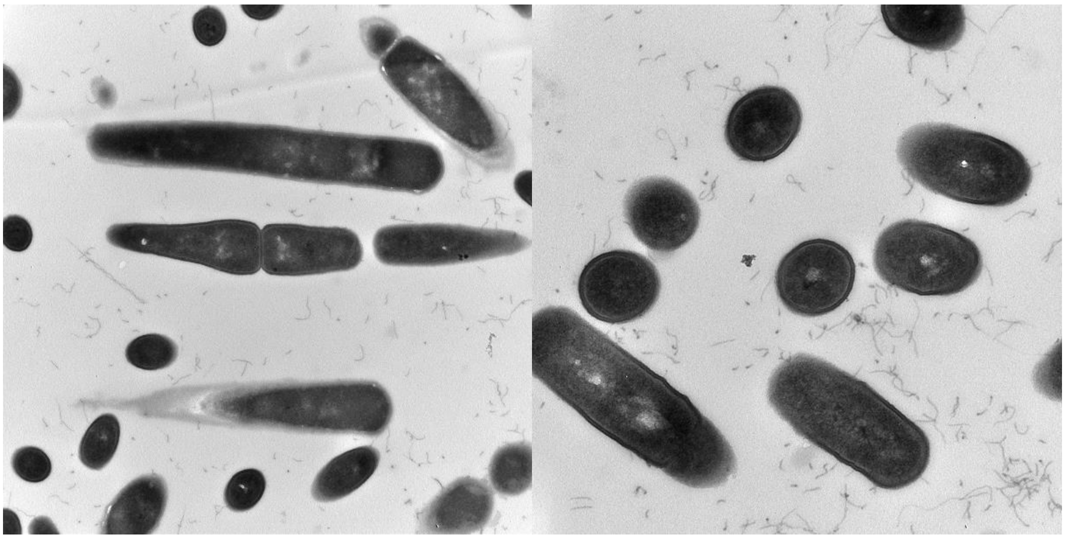

(**a**) (**b**)

Figure S2. TEM images of *C. michiganensis* (a) bacteria treated with alterperylenol (**4**) at concentration of 4×MIC for 12 h; (b) untreated bacteria

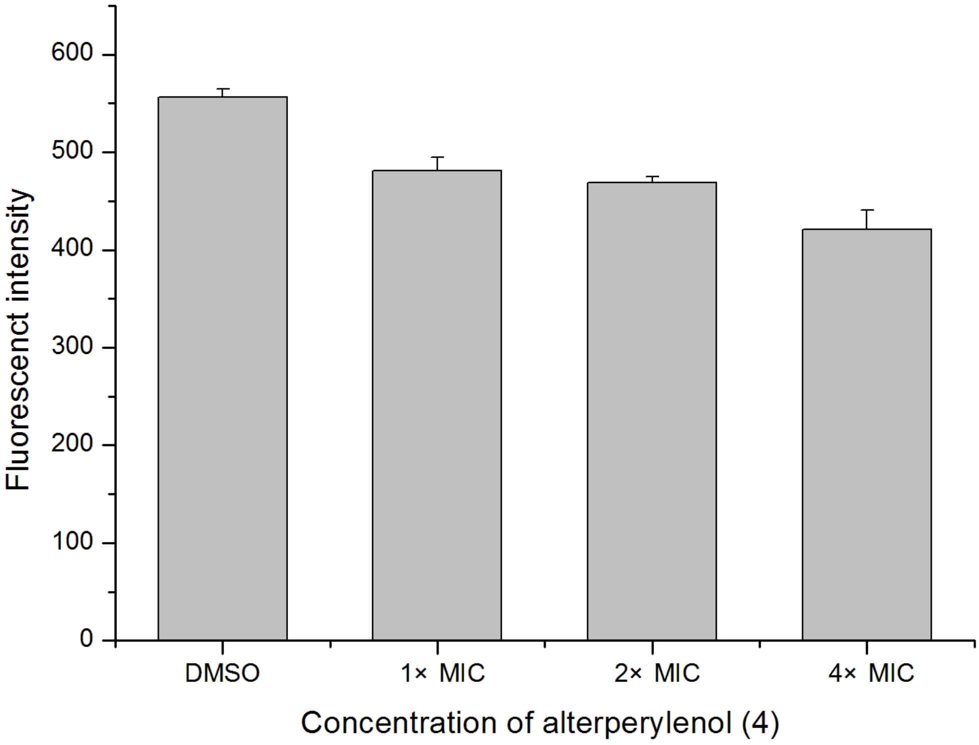

Figure S3. Effect of alterperylenol (**4**) on the membrane potential of *C. michiganensis*

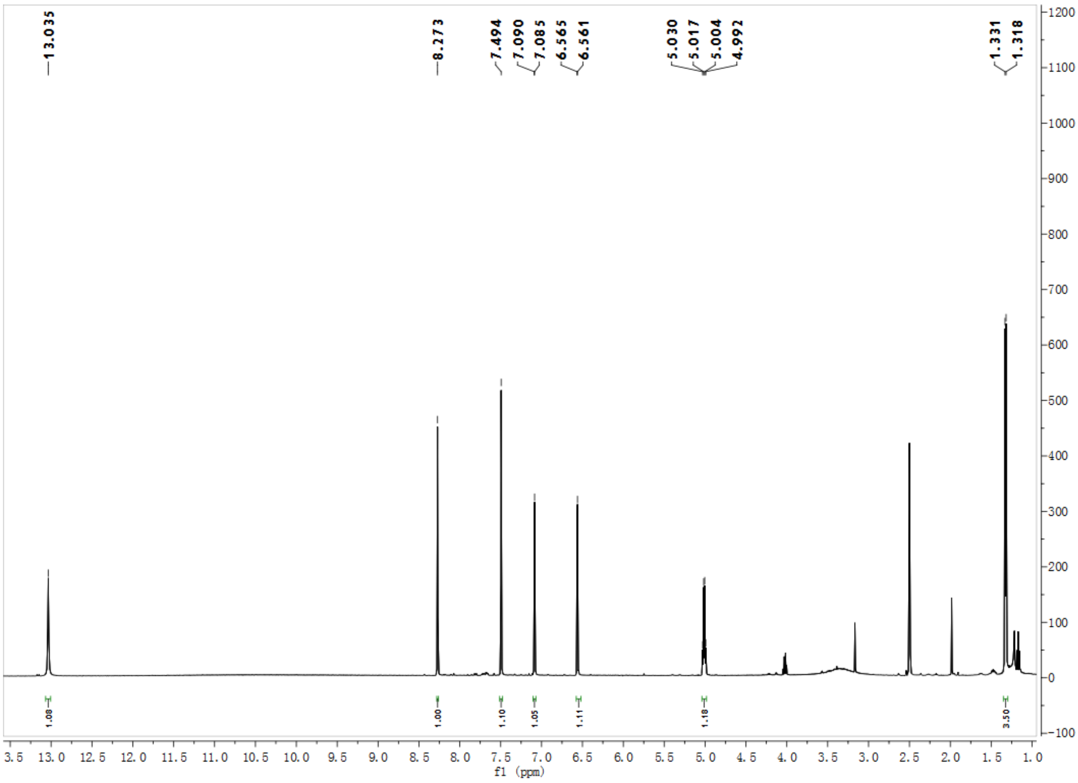

Figure S4. ^{1}H NMR (500 MHz, DMSO) spectrum of compound **1**

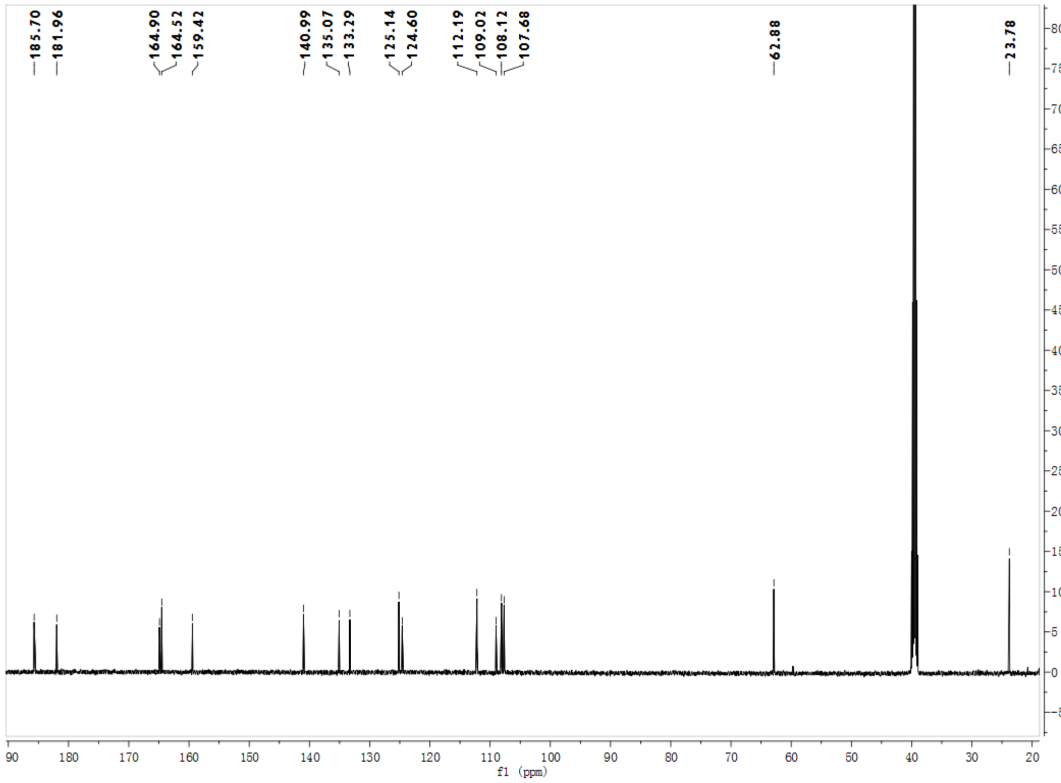

Figure S5. ^{13}C NMR (125 MHz, DMSO) spectrum of compound **1**

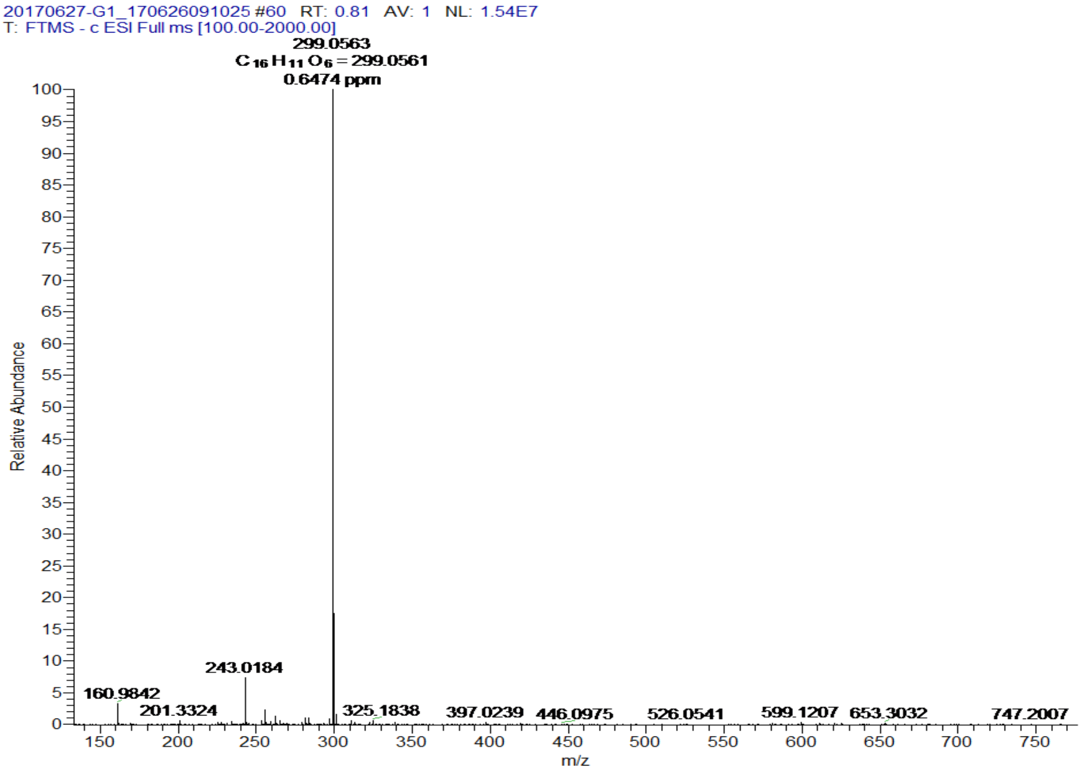

Figure S6. HRESIMS spectrum of compound **1**

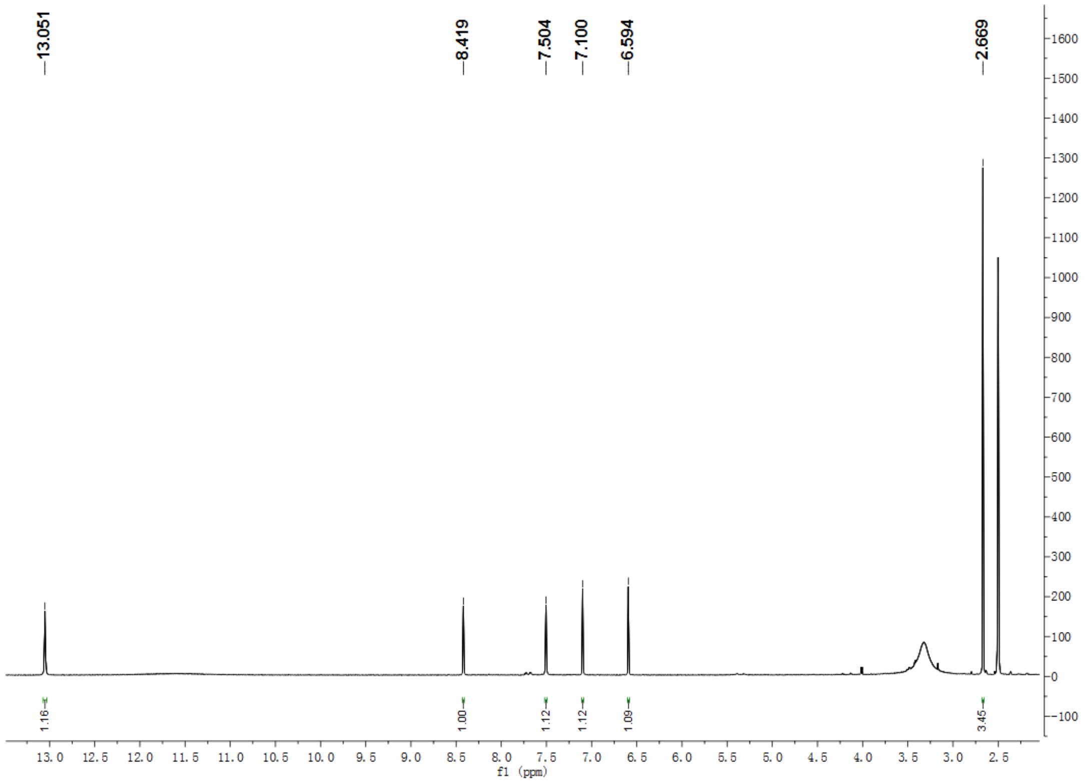

Figure S7. ^{1}H NMR (500 MHz, DMSO) spectrum of compound **2**

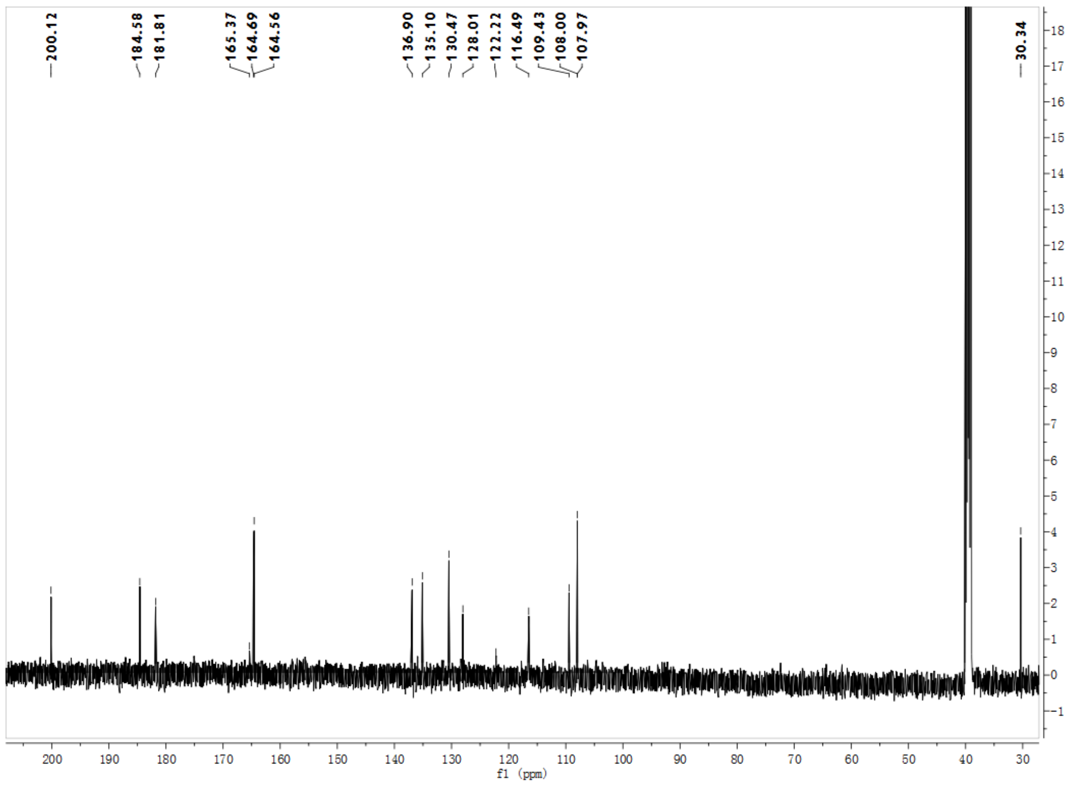

Figure S8. ^{13}C NMR (125 MHz, DMSO) spectrum of compound **2**

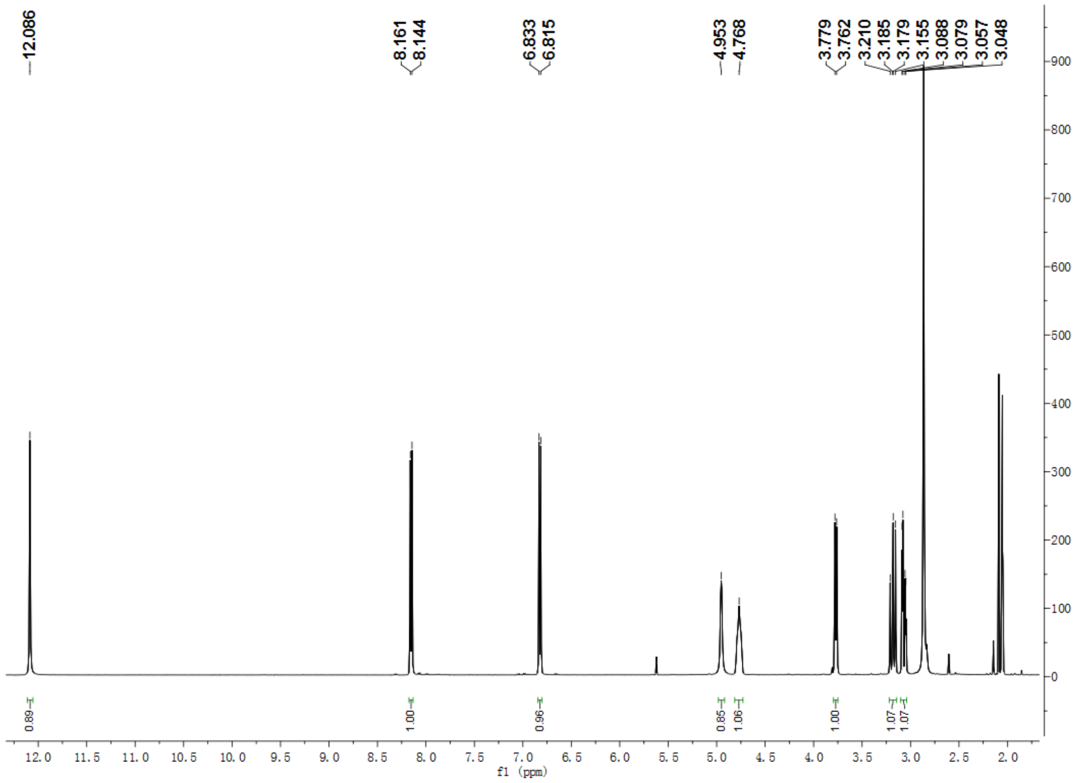

Figure S9. ^{1}H NMR (500 MHz, acetone-d_6) spectrum of compound **3**

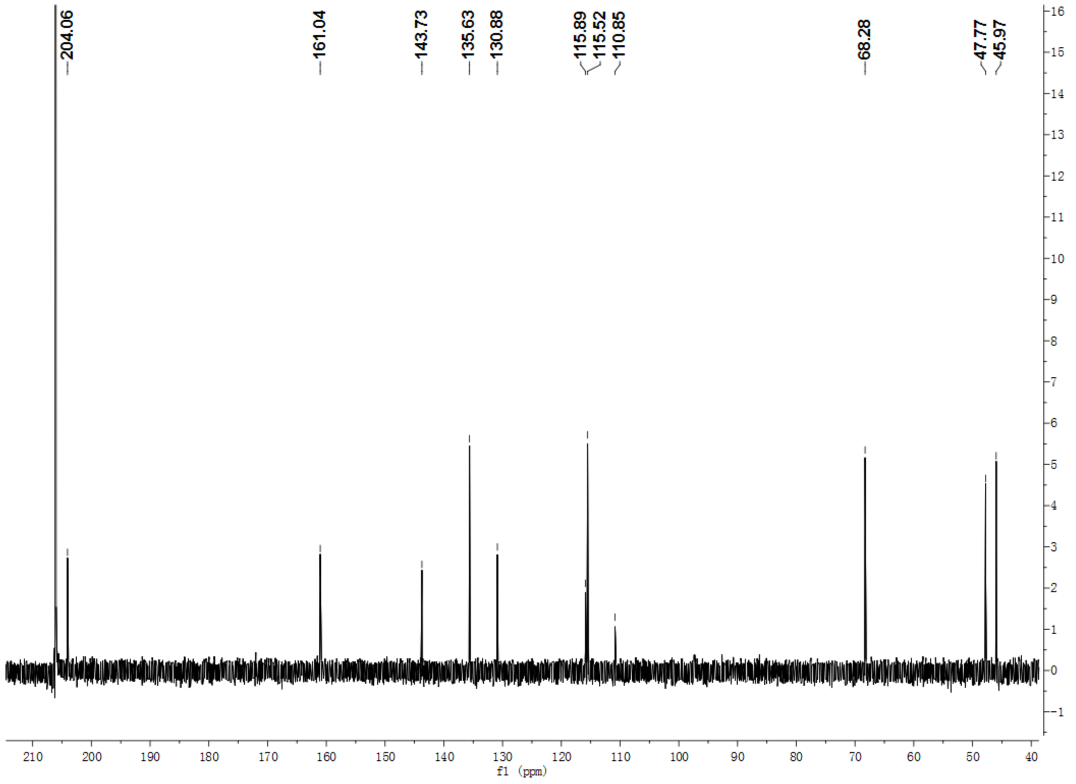

Figure S10. ^{13}C NMR (125 MHz, acetone-d_6) spectrum of compound **3**

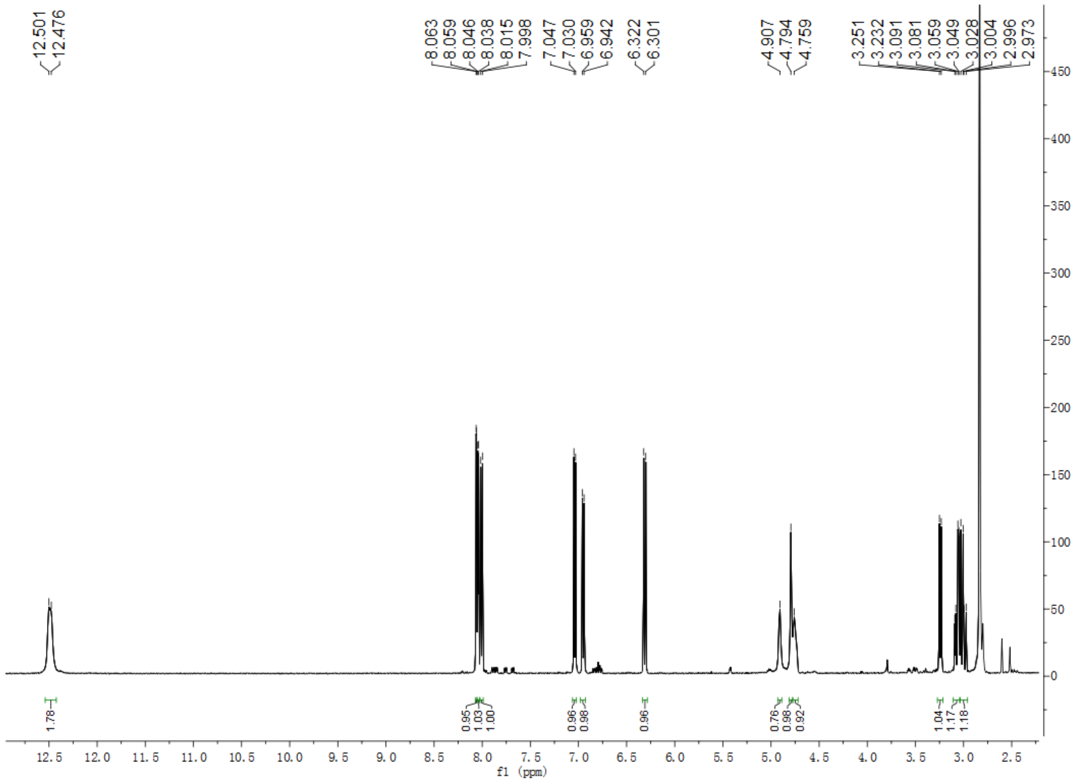

Figure S11. ^{1}H NMR (500 MHz, acetone-d_6) spectrum of compound **4**

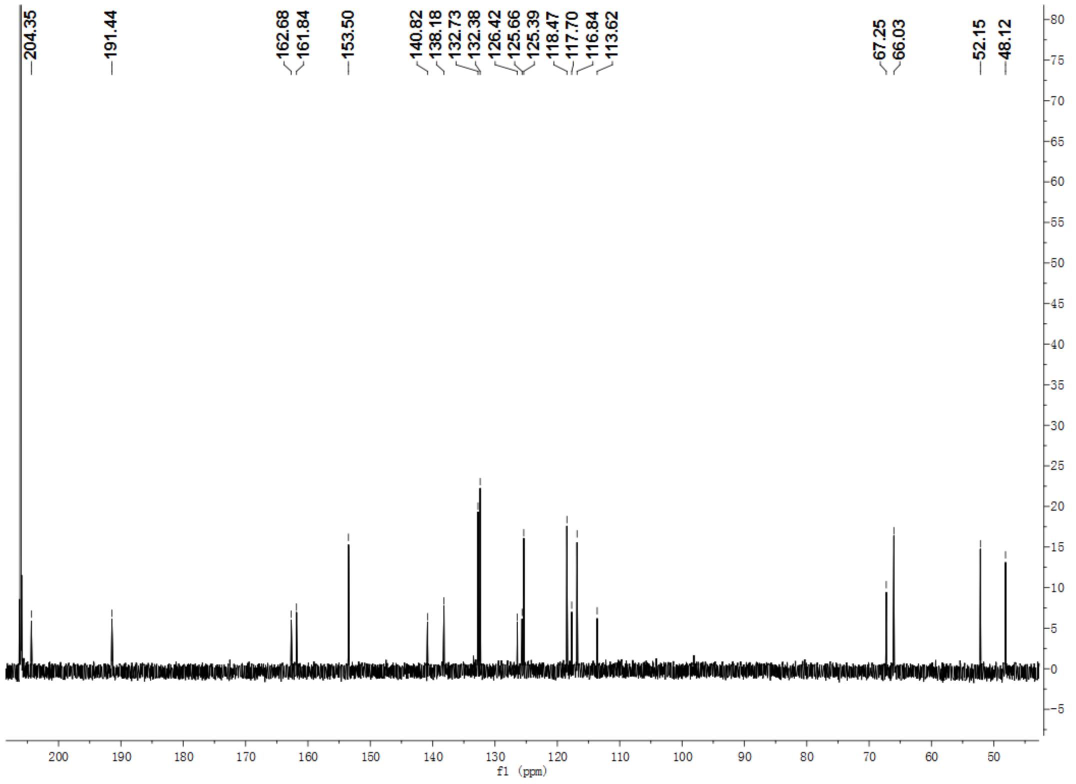

Figure S12. ^{13}C NMR (125 MHz, acetone-d_6) spectrum of compound **4**

Conf.1

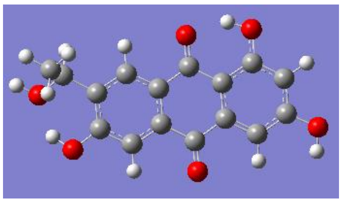

G = -1068.640548

Conf.2

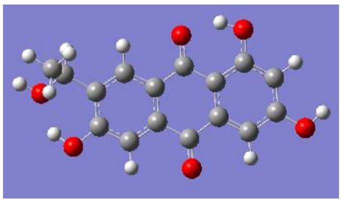

G = -1068.639944

Conf.3

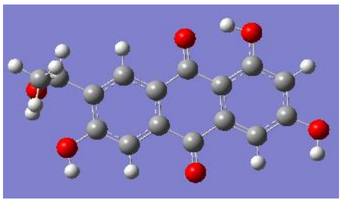

G= -1068.635597

Conf.4

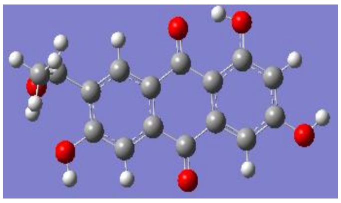

G = -1068.635219

Conf.5

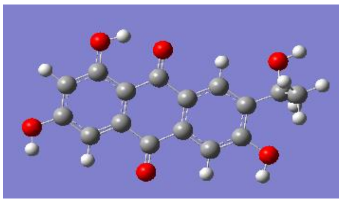

G= -1068.634926

Conf.6

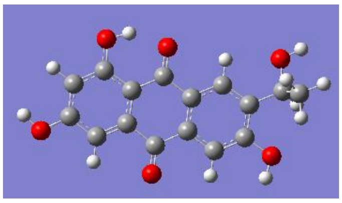

G = -1068.634524

Conf.7

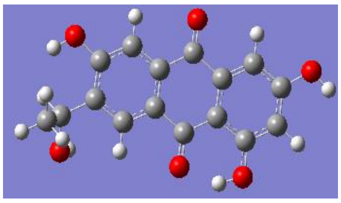

G = -1068.632124

Conf.8

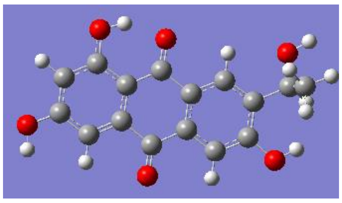

G = -1068.631064

Conf.9

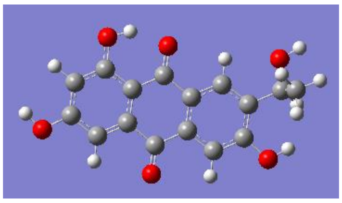

G = -1068.630497

Conf.10

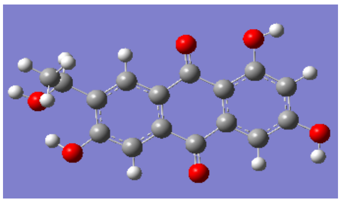

G = -1068.618785

Figure S13. Lowest energy conformers, G (B3LYP/6-311++G(2d,p) energy)
